# Five Years Follow-up of Imlifidase Desensitized Kidney Transplant Recipients

**DOI:** 10.3389/ti.2025.15425

**Published:** 2025-11-27

**Authors:** Tomas Lorant, Bonnie E. Lonze, Robert A. Montgomery, Niraj M. Desai, Christophe Legendre, Torbjörn Lundgren, Bengt von Zur-Mühlen, Ashley A. Vo, Kristoffer Sjöholm, Anna Runström, Jan Tollemar, Stanley C. Jordan

**Affiliations:** 1 Department of Surgical Sciences, Section of Transplant Surgery, Uppsala University, Uppsala, Sweden; 2 Department of Surgery, NYU Langone Transplant Institute, New York, NY, United States; 3 Northwell Transplant Institute, Manhassett, NY, United States; 4 Nephrology and Transplantation Unit, Hôpital Necker and Université Paris Cité, Paris, France; 5 Department of Transplantation Surgery, Karolinska Institutet, Stockholm, Sweden; 6 Cedars-Sinai Medical Center, Comprehensive Transplant Center, Los Angeles, CA, United States; 7 Hansa Biopharma AB, Lund, Sweden

**Keywords:** desensitization, highly sensitized patients, imlifidase, kidney transplantation, long-term follow-up

## Abstract

In four phase 2 clinical trials, patients underwent imlifidase-enabled kidney transplantation, converting a positive crossmatch (+XM) to negative. Here, we present data from 39 patients enrolled in the 5-year follow-up extension trial. Patient survival, graft survival, renal function, delayed graft function (DGF) antibody mediated rejection (AMR) frequency, safety and presence of donor specific antibodies (DSAs) are presented. Data from the long-term follow-up trial (17-HMedIdeS-14); NCT 03611621) with planned visits at 1, 2, 3, and 5 years after imlifidase were combined with up to 6-month phase 2 data from 4 pivotal trials (13-HMedIdeS-02, 13-HMedIdeS-03, 14-HMedIdeS-04, 15-HMedIdeS-06). Five-year patient survival was 90% with 3 deaths occurring between 6 months and 1 year, with no deaths occurring subsequently. Death-censored graft survival was 82% (with 3 early graft losses and 3 graft losses between 2 and 5 years). Mean eGFR at 5 years was 50.1 (MDRD), 55.8 (CKD-EPI, 2021) and 52.5 (KRS, 2023) mL/min/1.73 m^2^ (N = 24 patients) among functioning grafts. DSA rebounded with levels progressively decreasing over time and no increase in DSA seen between 3 and 5 years. No AMR occurred between 3 and 5 years. Furthermore, no additional safety signals assessed as related to imlifidase were reported in this cohort. At 5 years, highly-HLA sensitized patients who underwent imlifidase desensitization prior to transplantation demonstrated excellent patient and graft survival, despite their high-risk immunological profile. Imlifidase offers a viable and effective treatment option for highly sensitized patients to achieve life-saving transplantation and continued optimism is warranted.

**Clinical Trial:**

ClinicalTrials.gov (NCT02790437), EudraCT Number: 2016-002064-13.

## Graphical Abstract

**Figure:**
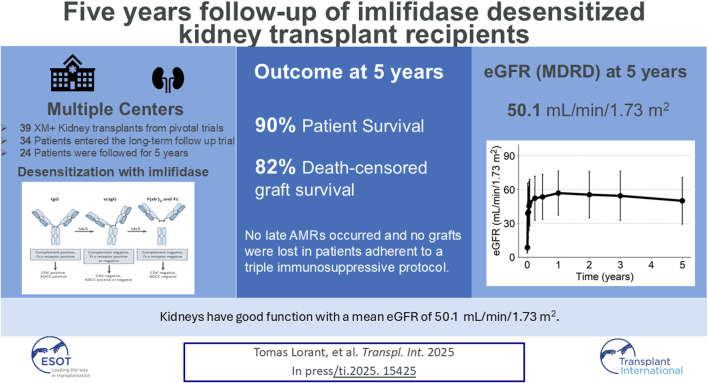

## Introduction

Kidney transplantation is the preferred treatment option for patients with end-stage renal disease, however, it is not readily accessible to the highly sensitized patient population [1–3]. Prioritization within kidney allocation systems, transplantation through acceptable antigen mismatch programs, and living donor paired exchange programs have led to substantial increases in compatible transplants for all but the most highly sensitized [3]. Desensitization has been utilized as a strategy to increase the opportunity for transplantation in sensitized candidates who are biologically incompatible with a high percentage of available donors and therefore remain difficult to transplant, despite the different strategies. However, transplantation rates for patients with cPRA ≥99.9% are so low that these patients are more likely to die or be removed from the transplant list than ever receiving a life-saving kidney transplant [1–3]. Since there is currently no standard of care for desensitization, patients endure extensive wait times, which increase morbidity and mortality. There is a clear and persistent need for therapies that can reduce or eliminate the immunologic barrier to transplantation posed by HLA sensitization. Current desensitization therapies often take months to years to even show minimal effects on HLA antibodies in the most sensitized patients with patients often needing multiple desensitization approaches [1–7]. In addition, the paucity of literature describing long-term outcomes of these patients, introduces uncertainty when evaluating the benefits of desensitization transplantation versus the potential risks of a higher immunologic risk transplants [6, 7].

Imlifidase is a cysteine proteinase derived from immunoglobulin G (IgG)-degrading enzyme of *Streptococcus pyogenes* (IdeS), which effectively cleaves all preformed IgG, including anti-HLA antibodies, intravascular and extravascular, inhibiting complement-dependent cytotoxicity (CDC) and antibody-dependent cellular cytotoxicity (ADCC) within hours of administration. Thus, imlifidase opens up an antibody-free window to enable safe HLA-incompatible transplantation from both living and deceased donors [8–13]. An analysis at 3 years of the crossmatch positive population has been published previously, reporting on 39 patients receiving living- or deceased-donor kidney transplants across four pivotal phase 2 studies conducted in the United States and Europe [13]. Three years after imlifidase-enabled desensitization and transplantation, death-censored allograft survival was 84%, patient survival was 90% and mean eGFR (MDRD) was 50.1 mL/min/1.73 m^2^. The results demonstrated a consistent safety profile with the highest risk of antibody mediated rejection (AMR) seen during the first weeks following imlifidase enabled transplantation. To date, imlifidase (Idefirix®, Hansa Biopharma AB, Lund, Sweden), remains the only conditionally regulatory approved agent in EU/EEA, Australia, Israel, Switzerland and the United Kingdom for desensitization in adult kidney transplantation.

Here we report results of a 5-year long-term follow-up study in which these imlifidase-desensitized kidney transplant patients enrolled (17-HMedIdeS-14; NCT 03611621).

## Materials and Methods

A 5-year analysis of patient survival, graft survival, renal function, AMR frequency, safety and presence of donor specific antibodies (DSAs) is presented. From 2014-2017, 46 adult (18–70 years) patients at 6 transplant centers (Sweden 2, France 1 and US 3) received an imlifidase-enabled transplantation across four phase 2 clinical trials, with data collected up to 6-month post-transplant. Upon completion of the initial studies, patients were asked to participate in this non-interventional long-term follow up (LTF) study, in which collected standard of care follow-up data were collected annually. The LTF study (17-HMedIdeS-14; NCT 03611621) had planned data collection at 1, 2, 3, and 5 years after imlifidase and was combined with the phase 2, 6-month data. Only patients with a positive crossmatch (+XM) to their donor kidney prior to imlifidase treatment were included in the final analysis (N = 39 out of 46). All studies were conducted in accordance with the ethical principles that have their origins in the Declaration of Helsinki; all ethical and regulatory approvals were available before any patient was exposed to any study-related procedure. Each study was reviewed and approved by authorities and each center’s institutional review board before study initiation with all patients providing written informed consent.

### Outcomes

Details of the methodology for clinical outcomes of patient survival, graft survival, graft function, DSA levels, anti-drug antibodies (ADA), HLA characteristics of the pooled analysis have been previously published [13]. Biopsy proven AMR was diagnosed according to Banff 2017 criteria. Estimated glomerular filtration rate (eGFR) was calculated from local serum creatinine measurements using three different methodologies: the four-variable modification of diet in renal disease, MDRD^1^, and the equations removing race as a variable, CDK-EPI (2021)^2^, as well as the KRS (2023)^3^

### Statistical Methods

Survival analysis was performed using Kaplan-Meier methodology. Patients were censored at the last know visit or death. In addition, censoring was performed for loss to follow-up and death with a functioning graft with death only depicted in the graft-loss figures. DSA and eGFR were calculated according to linear model from 3 months up to 5 years. All statistical analyses were performed using R version 4.2.1 (R Core Team (2018), R Foundation for Statistical Computing, Vienna, Austria).

## Results

### Demographic and Baseline Characteristics

The four pivotal trials included 46 patients, among which 39 were XM+ and included in the analysis. Subsequent or prior to completion of the parent trial, 12 patients did not actively enroll in the follow-up trial; 3 patients had died, 3 patients suffered graft loss in parent trial, 2 declined to participate in the LTF study and 4 could not be contacted. During years 2–5, 3 patients lost their grafts thus 24 patients could be followed up to 5 years.

The study population was predominantly Caucasian (77%), from the US (72%), EU (28%) who received a deceased or living donor kidney (DD, 82% and LD, 18%) (Supplementary Table S1). Mean age at transplant was 43.2 years (SD 13), with 69% of patients having had at least one transplant prior to imlifidase desensitization. Mean time on dialysis prior to the current transplant was 6.4 years (SD 5.6). All patients were highly sensitized, with a median cPRA of 99.62% (range 41.67%–100%).

### Clinical Assessments

**FIGURE 1:**
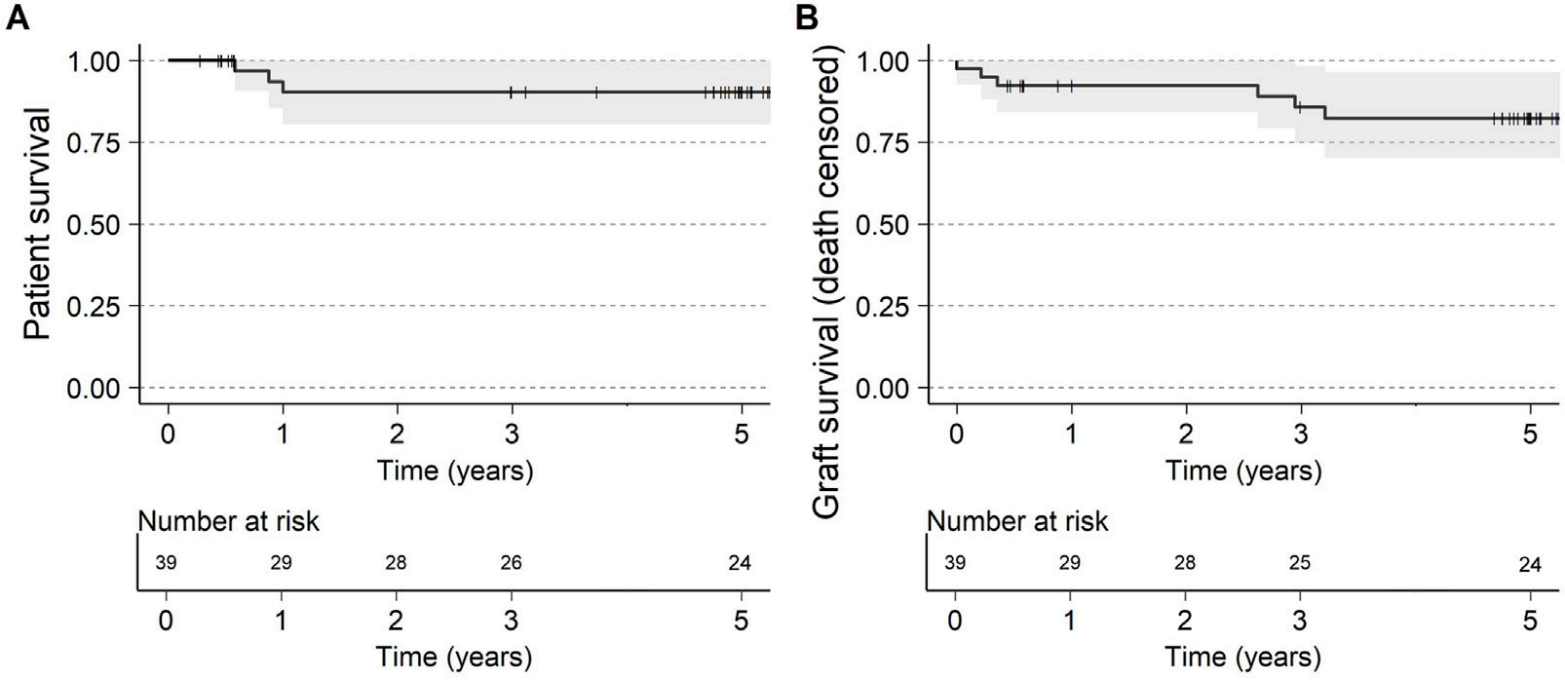
**(A)** Patient survival and **(B)** Graft survival (death censored) up to 5 years after transplantation. Visit window of the 5-year visit was ±6 months.

Three deaths occurred between 6 months and 1 year (one death was attributed to *pseudomonas* bacteremia/influenza, one due to cardiac arrest; while the cause of the third death was unknown), with no deaths occurring between 1 and 5 years. The 5-year patient survival was 90% (CI: 80%–100%, Figure 1A). No deaths were attributed to imlifidase or to graft failure.

Death censored graft survival at 5-year was 82% (CI: 70%–96%, Figure 1B) with 3 graft losses early in the studies attributed to one IgM mediated hyperacute rejection and two primary non-functioning grafts. No grafts were lost due to AMR throughout the trial period and DGF in patients with AMR was 47% compared to 42% in XM+ patients without AMR (Supplementary Table S1).

**FIGURE 2:**
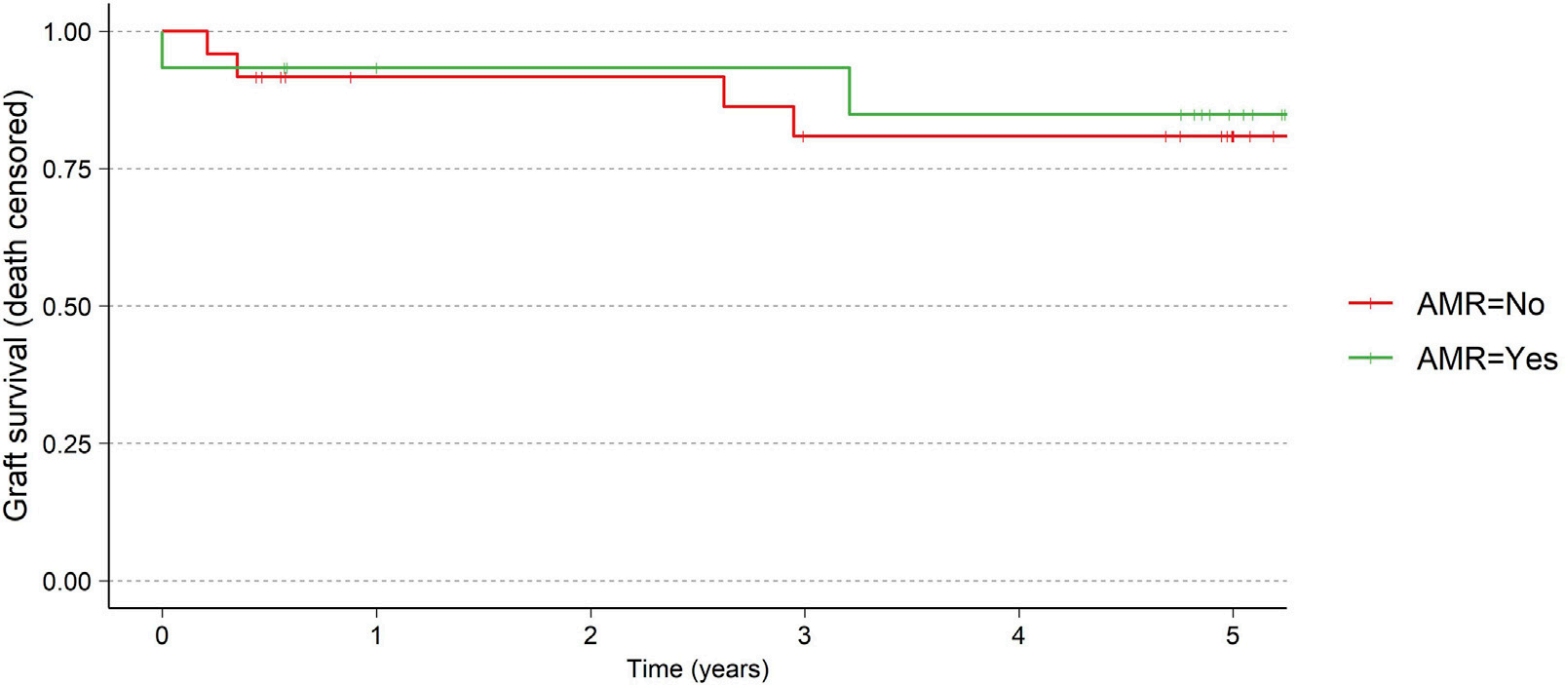
Graft survival for AMR vs. no-AMR.

There was no difference in graft survival (death censored) between the patients experiencing AMR and the patient without (Figure 2). Two allograft losses occurred between 2 and 3 years, attributed to reduction of immunosuppression secondary to an infection in one patient and immunosuppression medication non-adherence in another. One graft loss occurred between 3 and 5 years due to a continuous decline in graft function over time from a transplant which initially struggled with acute tubular necrosis and delayed graft function. Data on 24 patients, was available who were followed for 5 years.

**FIGURE 3:**
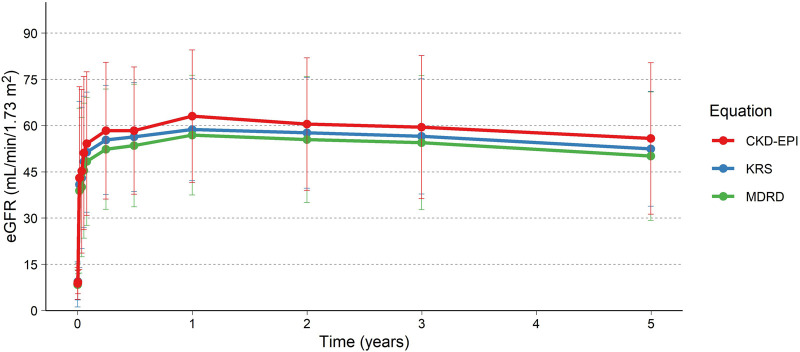
eGFR up to 5 years using the MDRD, CKD-EPI and KRS (2023). Colored lines and points are the mean, and the error bars are the standard deviation. Only subjects with measurements after the initial studies, i.e., at 1 year or later, included in the analysis (N = 25).

**FIGURE 4:**
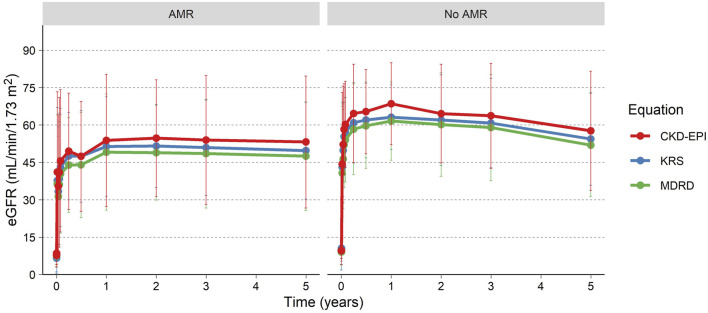
eGFR over time for patients experiencing an AMR and patients without AMR.

eGFR for the patients is presented in Table 1; Figure 3, using the MDRD, the CKD-EPI (2021) and the KRS equations. For patients with a functioning graft and with available data, mean eGFR estimation at 5 years was 50.1 (MDRD), and 55.8 (CKD-EPI, 2021), and 52.5 (KRS) mL/min/1.73 m^2^ for the three equations respectively. At 5 years 79% (MDRD) of patients had an eGFR ≥30 mL/min/1.73 m^2^. The slope of the eGFR remained stable over time with an initial increase seen earlier in the transplantation course (Figure 3). The eGFR did not differ significantly at the 5-year visit between patients who experienced AMR compared to patients without AMR (Figure 4; Table 2).

**TABLE 1:** eGFR up to 5 years using MDRD, CKD-EPI (2021) and KRS (2023).

Timepoint	eGFR (mL/min/1.73 m^2^)			iDSA (immunodominant DSA, MFI)		
	MDRD Mean (SD)	CKD-EPI Mean (SD)	KRS Mean (SD)	Immunodominant DSA Mean (SD)	Class I Mean (SD)	Class II Mean (SD)
Predose and Tx	8.4 (4.8), n = 22	8.8 (5.1), n = 22	8.5 (7.3), n = 22	9,772.7 (7,173.9), n = 22	8,884.7 (6,806.1), n = 10	10,512.7 (7,683.5), n = 12
Month 6	53.5 (19.9), n = 23	58.4 (20.6), n = 23	56.3 (17.7), n = 23	5,420.8 (6,247.3), n = 20	2,499.6 (2,164.1), n = 9	7,810.9 (7,513), n = 11
Year 1	56.9 (19.4), n = 24	63.1 (21.5), n = 24	58.7 (16.6), n = 24	1,427.3 (1,026.7), n = 6	1,886 (267.3), n = 2	1,198 (1,234), n = 4
Year 2	55.5 (20.5), n = 24	60.5 (21.5), n = 24	57.7 (18.0), n = 24	4,209.2 (4,285.3), n = 12	731 (661.1), n = 3	5,368.7 (4,369.4), n = 9
Year 3	54.5 (21.7), n = 23	59.5 (23.2), n = 23	56.5 (18.7), n = 23	1,783.2 (2,254.5), n = 17	907.2 (532.8), n = 8	2,561.9 (2,910), n = 9
Year 5	50.1 (20.8), n = 24	55.8 (24.5), n = 24	52.5 (18.7), n = 24	2,522.6 (4,379.3), n = 15	543.3 (289), n = 6	3,842.1 (5,349), n = 9

DSA is evaluated as the Immunodominant DSA and separated by the class of the immunodominant DSA. Presented in the table are mean, standard deviation and the number of subjects evaluated at each timepoint and analysis.

**TABLE 2:** eGFR at 5 years after transplantation for patients experiencing AMR vs. no-AMR.

eGFR equation (mean [SD])	AMR (N = 10)	No AMR (N = 14)	P-value
MDRD	47.5 (21.8)	52 (20.7)	0.546
CKD-EPI	53.2 (26.5)	57.7 (23.9)	0.703
KRS	49.8 (19.4)	54.4 (18.6)	0.667

P-value calculated using Mann-Whitney U test.

**FIGURE 5:**
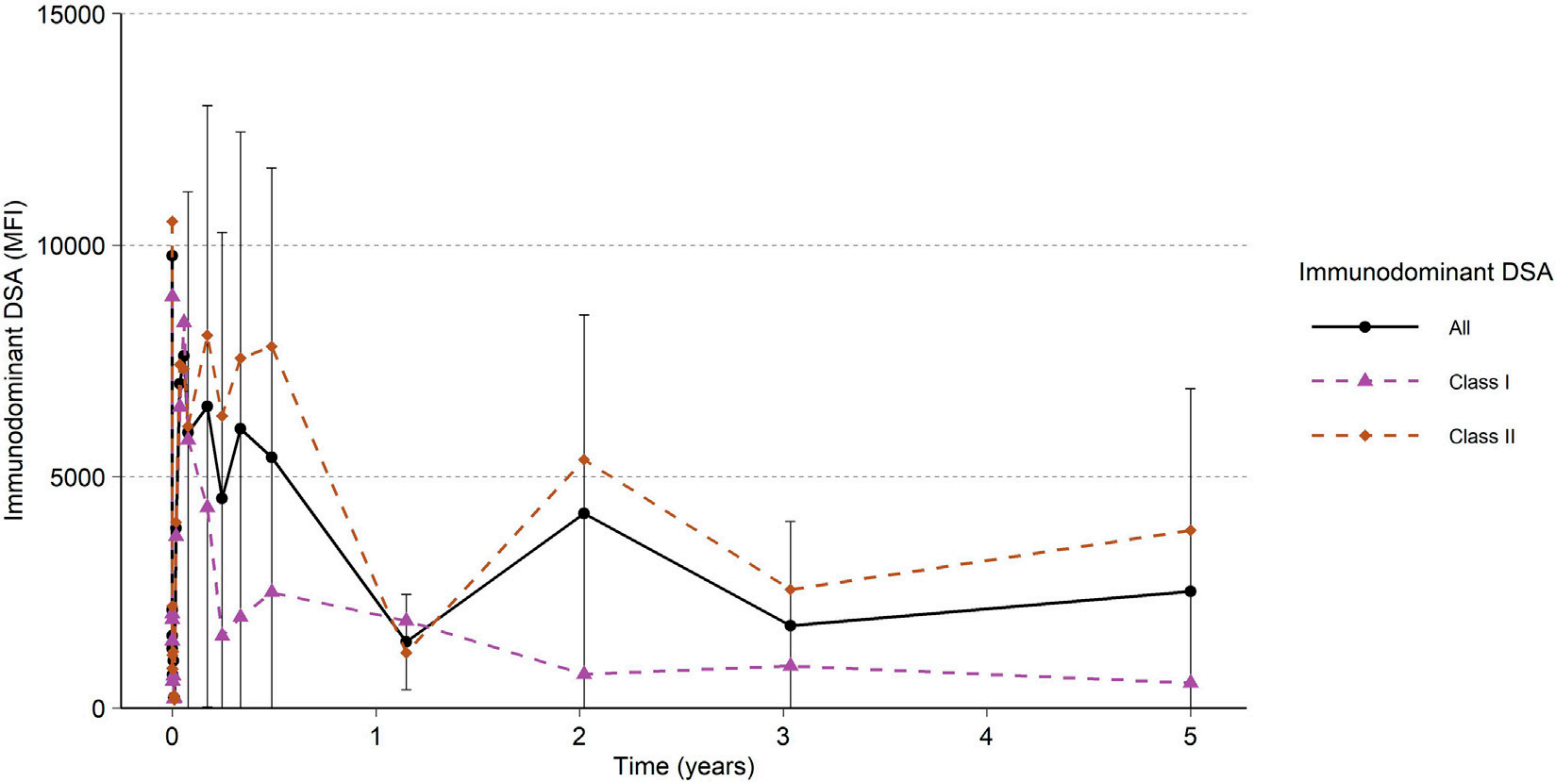
All Immunodominant DSA and Immunodominant DSA per HLA class I and HLA class II. Black and colored lines and points are the mean, and the error bars are the standard deviation. Only subjects with centrally analyzed samples after the initial studies, i.e., at 1 year or later, included in the analysis (N = 22).

Occurrence of DSA at 5 years was 27% and was defined as any DSA with an MFI ≥2,000 (Table 1). As previously described, the MFI remained low for approximately 1 week after imlifidase treatment, then rebounded to approximately 80% of pre-treatment levels, with the peak occurring 14 days post-treatment (Figure 5). Immunodominant Class II DSA was more prevalent than Class I. In general, the Class II DSA strength was higher pre-dose and with rebound after imlifidase dosing (Figure 5). After initial peak at 14 days post-treatment, the levels of DSA progressively decreased over time, which was sustained at 5 years with an MFI for Class I DSA below 1,000 and below 4,000 for Class II DSA. Patients who demonstrated no DSA at 6 months remained DSA rebound-free during the 5-year follow up. Only one patient developed *de novo* DSA during the study. DSA levels did not differ significantly at the 5-year visit between patients that experienced AMR compared to patients without AMR, with a median (range) of 1,838 (348; 17,630) MFI and 948 (258; 4,130) MFI, respectively. This patient with *de novo* DSA developed these antibodies after an infection 3-year post-transplant which required reduction in immunosuppression, and unfortunately the graft was subsequently lost.

### Antibody Mediated Rejection (AMR)

Allograft rejection was required to fulfill the criteria outlined in the Banff 2017 criteria throughout the assessed period (biopsy taken at time of graft rejection episode, histological evidence of AMR on pathology and presence of detectable DSA). The majority of AMR occurred within the first month after transplantation (N = 11), with 4 additional AMRs recorded between 2 and 6 months from transplantation totaling an AMR rate in the first 6 months post-transplantation of 38%. In the extended follow up, 7 rejection episodes were reported, with 5 clinically suspected allograft rejections that did not meet study criteria (Banff 2017); one at 18 months post-transplant that was C4d negative/DSA negative, one at 24 months post-transplant that was C4d negative/DSA negative with pathology that did not meet criteria, one at 45 months post-transplant that was DSA negative and with inadequate biopsy for diagnosis, one at 57 months post-transplant diagnosed with no biopsy proven acute rejection (BPAR) with no additional data provided, and one at 60 months that was C4d negative/DSA negative which was not treated. Therefore, all histologically confirmed AMRs remained from the timeframe of the first few months following transplantation and were diagnosed and treated with standard available therapies as described previously [13]. The one biopsy confirmed acute rejection was recorded 9 months after transplantation and was a previous AMR recorded in the phase 2 trial. To date, no allograft losses attributable to AMR have been reported.

### Safety

Adverse events assessed as related to imlifidase have been previously described and remain unchanged [10–13]. At 5 years, no additional safety signals assessed as related to imlifidase have been reported.

Comorbidities in the form of infections were reported in 18 of the 37 patients enrolled. The most common followed for 5 years, were urinary tract infection (37 occasions reported in 9 patients), nasopharyngitis (10 occasions reported in 3 patients), pyelonephritis (6 occasions reported in 3 patients), upper respiratory infection (5 occasions reported in 4 patients), and COVID-19 infection (4 occasions reported in 4 patients). In addition, 2 patients reported 6 events of urosepsis, 2 patients reported 2 events of sepsis, and 2 patients reported 2 events of pneumonia. No death or graft loss occurred due to infection beyond 6 months after transplantation. No BK virus infections were reported up to 5 years follow-up and 23/24 tested negative in PCR evaluation (<500 copies) at 5 years. There have been no reported malignancies in the patients during the long-term follow-up (data on file).

## Discussion

Kidney allocation systems are continually being reviewed and refined to balance both equity and utility for the scarce resource of deceased donor kidneys, with efforts to prioritize sensitized candidates when a compatible donor organ becomes available [14–19]. What remains consistent is a cohort of the most highly sensitized patients whose statistical probability of finding a compatible offer is so low, that desensitization to facilitate an HLA-incompatible transplant is the only path to the survival benefit afforded by transplantation instead of dialysis.

The ability of imlifidase to effectively cleave and reduce the entire spectrum of preformed IgG anti-HLA antibodies, regardless of intensity or subclass, might open up a new era in desensitization in transplantation [20, 21]. Compared to other desensitization methods, imlifidase provides within hours a near complete IgG antibody-free window, even in patients with positive cytotoxic crossmatches, and thus enables transplantation in both the living and deceased donor settings [22, 23]. Initially some expressed reservation regarding the broader use of imlifidase and incompatible transplantation due to speculation of early immunologically driven graft losses. Furthermore, the risk of increased cold ischemia time and thereby risk of higher DGF due to crossmatch evaluation before and after treatment, has been mentioned as a potential risk. DGF impact on long-term function remains unclear. However, in the present 5 years pooled data analysis this seems unlikely, as imlifidase-enabled transplant recipients continue to demonstrate sustained patient and graft survival with relative stability of allograft function despite the high-risk immunological profile, and in whom transplant may have not been possible otherwise.

Of the patients with a functioning graft and available data at 5 years, 79% had an eGFR above 30 mL/min/m^2^ with a mean eGFR of 50.1 mL/min/1.73 m^2^ (calculated by MDRD). Guidelines supporting the use of the most recent race-free transplant specific eGFR equation, eGFR calculations were also provided using the CKD-EPI (2021) and KRS equations for comparison [24, 25]. We believe this finding is important as many of the advantages of transplantation over dialysis are linked to adequacy of kidney allograft function and are lost with compromised eGFR [26–29]. In the current data, eGFR continues to be sustained, indicating low likelihood of chronic kidney disease progression or impending graft failure. The incidence of AMR was similar to those reported at 3 years and may be related to the overall pattern of declining DSA over time across both class I and class II antibodies. This is most probably due to continuous good immunosuppressive therapy, not triggering immunologic response and DSA increase which could be seen in a patient where immunosuppression was reduced, due to severe infection. Data up to 21 days post-transplantation confirms that when a rejection occurs, DSA rises during the early period of DSA rebound thus indicating AMR in a noninvasive test. The frequency or severity of early AMR was not substantially different from what is expected and reported in highly sensitized candidates receiving incompatible kidneys and few late AMRs were reported. Patients’ AMR were treated with available standard available therapies, and no grafts were lost due to AMR. The current data indicate that the challenge of AMR in these patients is manageable, and no greater with imlifidase desensitization than with other approaches to transplantation in this at-risk group of highly sensitized patients. The long-term safety profile of imlifidase was consistent with that described previously with no increase in safety signals as it relates to infection or malignancy over time (data on file).

Limitations of this 5-year analysis of four 6-month pivotal trials are the lack of following relevant kidney function tests such as proteinuria and lack of long-term protocol biopsies. Although early AMRs were diagnosed and treated early and successfully, this does not preclude the presence of chronic active AMR (caAMR) and the incidence of this remains largely unknown over the time studied. Clinical parameters of interest to understand granular details of renal function over time including presence of proteinuria, and levels and type of immunosuppression were not part of the original trial design as long-term management of allografts will vary and are subject to much heterogenicity in real-world settings. An additional limitation is the loss of data over time. As common with long-term follow up studies, attrition will inevitably occur and unfortunately, data for some outcomes of interest were unavailable for all patients.

In conclusion, efficacy and safety outcomes were assessed in imlifidase treated kidney-transplant recipients at 5 years after treatment. Data remains the best available long-term data in a cohort of patients who received a crossmatch positive, incompatible kidney at the time of transplantation. Imlifidase demonstrated outcomes at 5 years that are comparable to those seen with compatible transplantation and indicates imlifidase represents an important new therapy to create a temporary “antibody-free window” enabling HLA incompatible transplantation. Some challenges remain associated with this desensitization methodology, in particular the risk of early antibody rebound, but this does not invariably occur in all patients, and with proactive management, it can be managed successfully. This 5-year LTF study suggests good long-term outcomes are achievable and are certainly superior to those for HLA sensitized patients who remained on dialysis [28].

## Data Availability

The original contributions presented in the study are included in the article/Supplementary Material, further inquiries can be directed to the corresponding author.

## Footnotes

1
https://www.acpjournals.org/doi/10.7326/0003-4819-145-4-200608150-00004?url_ver=Z39.88-2003&rfr_id=ori:rid:crossref.org&rfr_dat=cr_pub%20%200pubmed

2
https://www.nejm.org/doi/full/10.1056/NEJMoa2102953

3
https://doi.org/10.1136/bmj-2022-073654
