# Supplementary material for: Five Years Follow-up of Imlifidase Desensitized Kidney Transplant Recipients
Source: Transpl Int. 2025 Nov 27;38:15425. doi: 10.3389/ti.2025.15425 (PMC12697064; doi:10.3389/ti.2025.15425)
Supplement: Supplementary file 1 [file Table1.docx]

**Table S1. Demographics and baseline characteristics of the patients transplanted after imlifidase treatment.**

| **Characteristics** | | **XM+, N=39** | **AMR & XM+, N=15** | **No AMR & XM+, N=24** | **XM+, DD and cPRA**  **≥ 99.9%, N=13** | **P-value ^F, G^** |
| --- | --- | --- | --- | --- | --- | --- |
| **Patient age (years); mean (SD)** | | 43.2 (13.0) | 44.5 (14.3) | 42.3 (12.3) | 45.3 (12.6) | 0.904^F^ |
| **Female; N (%)** | | 18 (46%) | 6 (40%) | 12 (50%) | 5 (38%) | 0.883^G^ |
| **Region, US; N (%)** | | 28 (72%) | 9 (60%) | 19 (79%) | 11 (85%) | 0.468^G^ |
| **Race; N (%)** | **White** | 30 (77%) | 11 (73%) | 19 (79%) | 9 (69%) | 0.998^G^ |
|  | **Black** | 4 (10%) | 2 (13%) | 2 (8%) | 2 (15%) |  |
|  | **Asian** | 3 (8%) | 1 (7%) | 2 (8%) | 1 (8%) |  |
|  | **Other** | 2 (5%) | 1 (7%) | 1 (4%) | 1 (8%) |  |
| **Time on dialysis prior to imlifidase transplantation (years); mean (SD)** | | 6.4 (5.6) | 7.4 (6.1) | 5.9 (5.4) | 9.3 (7.2) | 0.370^F^ |
| **Deceased Donor; N (%)** | | 32 (82%) | 13 (87%) | 19 (79%) | 13 (100%) | 0.399^G^ |
| **Total CIT; mean (SD)** | | 21.0 (10.0) | 23.8 (11.5) | 19 (8.5) | 22.7 (9.6) | 0.554^F^ |
| **Re-transplants; N (%)** | | 27 (69%) | 10 (67%) | 17 (71%) | 9 (69%) | 1.000^G^ |
| **cPRA^A^ (%);  median (1^st^ & 3^rd^ quartile)** | | 99.62  (94.92, 99.96) | 99.80  (93.70,  99.99) | 99.53  (96.55,  99.91) | 99.99  (99.97, 100) | 0.345^F^ |
| **Crossmatch positive; N (%)** | | 39 (100%) | 15 (100%) | 24 (100%) | 13 (100%) | NA |
| **Pre-dose DSA^B^ (MFI);  median (1^st^ & 3^rd^ quartile)** | | 7,791  (4,108, 16,320) | 13,009  (6,515,  21,580) | 5,727  (2,699,  9,470) | 16,292  (7,133, 2,1824) | 0.027^F^ |
| **Pre-transplant DSA^C^ (MFI);  median (1^st^ & 3^rd^ quartile)** | | 774 (292, 1,754) | 1,584 (904-3303) | 576 (193-1387) | 1,292 (774, 26,00) | 0.032^F^ |
| **DGF^D^; N (%)** | | 17 (44%) | 7 (47%) | 10 (42%) | 6 (46%) | 1.000^G^ |
| **DGF duration^E^ (days);  median (1^st^ & 3^rd^ quartile)** | | 10 (6, 26) | 24 (8, 28) | 9 (4, 14) | 12 (9, 23) | 0.332^F^ |

^A^ Central analysis, cut-off set to 3,000 MFI

^B^ Immunodominant DSA, central analysis

^C^ Closest timepoint prior to transplantation, immunodominant DSA, central analysis

^D^ DGF defined as the need for dialysis first week post-transplant (primary non-functioning grafts included)

^E^ Non-functioning grafts counted as infinite

^F^ For continuous data ANOVA was used for p-value determination between XM+ and XM+, DD and cPRA and ≥ 99.9%

^G^ For categorical data Fisher’s exact test was used for p-value determination between XM+ and XM+, DD and cPRA and ≥ 99.9%
